# Development and Validation of a Realistic Neonatal Intestinal Jejunoileal Atresia Simulator for the Training of Pediatric Surgeons

**DOI:** 10.3390/children11091109

**Published:** 2024-09-11

**Authors:** Javier Arredondo Montero, Blanca Paola Pérez Riveros, Oscar Emilio Bueso Asfura, Nerea Martín Calvo, Francisco Javier Pueyo, Nicolás López de Aguileta Castaño

**Affiliations:** 1 Pediatric Surgery Department, Complejo Asistencial Universitario de León, 24008 León, Spain; 2 Department of Preventive Medicine and Public Health, School of Medicine, University of Navarra, 31008 Pamplona, Spain; 3 IdiSNA, Instituto de Investigación Sanitaria de Navarra, 31008 Pamplona, Spain; 4 CIBER de Fisiopatología de la Obesidad y la Nutrición, Instituto de Salud Carlos III, 28029 Madrid, Spain; 5 Department of Anesthesiology, University of Navarra Clinic, 31008 Pamplona, Spain; 6 Medical Engineering Laboratory, School of Medicine, University of Navarra, 31008 Pamplona, Spain

**Keywords:** intestinal atresia, jejunoileal atresia, simulation, silicone, 3D, pediatric surgery, training, model, open surgery

## Abstract

**Background:** Neonatal surgical pathology presents highly technical complexity and few opportunities for training. Many of the neonatal surgical entities are not replicable in animal models. Realistic 3D models are a cost-effective and efficient alternative for training new generations of pediatric surgeons. **Methods:** We conceptualized, designed, and produced an anatomically realistic model for the open correction of jejunoileal atresia. We validated it with two groups of participants (experts and non-experts) through face, construct, and content validity questionnaires. **Results**: The model was validated by eleven experts and nine non-experts. The mean procedure time for the experts and non-experts groups was 41 and 42 min, respectively. Six non-experts and one expert did not complete the procedure by the designed time (45 min) (*p* = 0.02). The mean score of face validity was 3.1 out of 4. Regarding construct validity, we found statistically significant differences between groups for the correct calculation of the section length of the antimesenteric border (Nixon’s technique) (*p* < 0.01). Concerning content validity, the mean score was 3.3 out of 4 in the experts group and 3.4 out of 4 in the non-experts group. **Conclusions:** The present model is a realistic and low-cost valid option for training for open correction of jejunoileal atresia. Before drawing definitive conclusions, future studies with larger sample sizes and blinded validators are needed.

## 1. Introduction

Pediatric Surgery is a highly complex surgical specialty that involves multiple organs, pathologies, and surgical procedures. In turn, the pediatric patient has unique differential characteristics, such as a lower homeostatic capacity and greater tissue fragility, which justifies the need for extreme delicacy and precision in the surgical act [1]. The training of new generations of pediatric surgeons is strongly conditioned by the possibility of acquiring and training in these complex and demanding technical competencies inherent to the specialty. While in many primary pathologies, the available volume is high and the learning curve is achievable [2], in other scenarios, the low prevalence of the disease conditions the training and learning possibilities.

Neonatal surgery, a significant part of the Pediatric Surgery specialty, exemplifies the above. Low European fertility [3] and recent remarkable improvements in prenatal diagnostic tools (which allow termination of pregnancy in cases of non-viability) [4] largely determine the volume of neonatal surgical pathology available. Lastly, the trend towards the use of minimally invasive techniques with complex and lengthy training curves (such as laparoscopy in congenital duodenal obstruction) [5] and the tendency to centralize cases in referral hospitals [6,7] make it difficult for new generations of residents to acquire the necessary surgical skills in these pathologies. Recent analyses confirm this decrease in index cases and highlight the need to reevaluate training programs and operative exposure in this specialty [8].

Given this situation, several options have been put forward to train new generations of pediatric surgeons: (1) The use of animal models. Multiple surgical training animal models presenting different pathologies have been previously reported. Notable pediatric examples are dismembered pyeloplasty surgery [9] and Swenson transanal endorectal pull-through [10]. Likewise, multiple animals are available depending on the required pathology and surgical anatomy [11]. Although animals have the great advantage of tissue realism and a mammal’s physiological and homeostatic conditions, they are expensive. Apart from that, many of the entities are not replicable in animal models because of their intrinsic characteristics (e.g., jejunoileal atresia, where there is a marked discordance in caliber between the intestinal ends, a situation that is not easy to replicate realistically in animal models). (2) The use of cadavers [12], which involves low availability, a high preparation cost, and a significant bioethical conflict, is not an acceptable resource for everyone. (3) Using simulation models built with different synthetic materials [13,14,15]. The marked industrialization and technological progression we are experiencing are contributing to lowering the design and production costs of these models and making their generalization for the training of pediatric surgeons possible. The most significant handicap of these models is, in many cases, the lack of realism. To the best of our knowledge, there is only one precedent in the literature regarding synthetic intestinal atresia models for the training of pediatric surgeons [16]. The present work aims to design, produce, and validate a low-cost and anatomically realistic model of neonatal jejunoileal atresia for the training of open corrective surgery by pediatric surgeons.

## 2. Methods

### 2.1. Conceptualization and Preliminary Design of the Model

**Figure 1:**
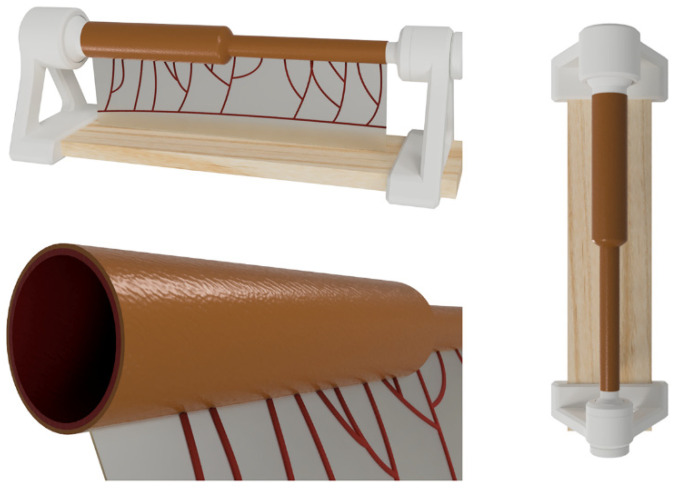
Images corresponding to the design phase of the model, made in Autodesk Fusion 360^®^ (Autodesk, CA, USA).

For the initial design, necropsic, surgical, and prenatal radiological references of the small bowel (normal, dilated/obstructed, and obliterated/defunctionalized) were obtained [16,17,18,19], and a preliminary range of measures and diameters was established. Autodesk Fusion 360^®^ (Autodesk, CA, USA) was used to design the model’s first iteration and establish an approximate proportional relationship between the two intestinal segments. Iterative adjustments and refinements were performed to ensure the model’s fidelity to neonatal bowel characteristics (Figure 1).

### 2.2. Model Production Methodology

The simulator comprises an anatomical model of jejunoileal atresia with discordant ends and a vascularized mesentery. The model is supported by a specially designed stand for proper usage.

To simulate the mucosa inside the intestine, platinum silicone Eco-Flex 0030 (Smooth-On) with red dye and Silicone Thinner (Smooth-On) additive in a 1A:1B:0.2C ratio were used. The mixture was injected into 3D-printed PLA molds using a Prusa MK3S 3D printer (Prusa Research, Prague, Czech Republic). After a 4-h curing period, the molded cylinder representing atresia was covered with a slightly larger mold to create the serosa layer. This serosa layer, composed of 15 g of Eco-Flex 0030 with a rosy pigment, was cured and introduced into a third mold to add the mesentery to the model. The mesentery was created using 5 g of Eco-Flex 0030 with a red pigment. An arbitrary arboriform vascular pattern was reproduced to simulate real vasculature. Finally, the model was coated with Silicone Thinner, an oily coating, to provide a more realistic experience. A custom-designed stand was manufactured to secure the model, allowing adjustment of rotation and tension without requiring external assistance.

### 2.3. The Final Version of the Model

**Figure 2:**
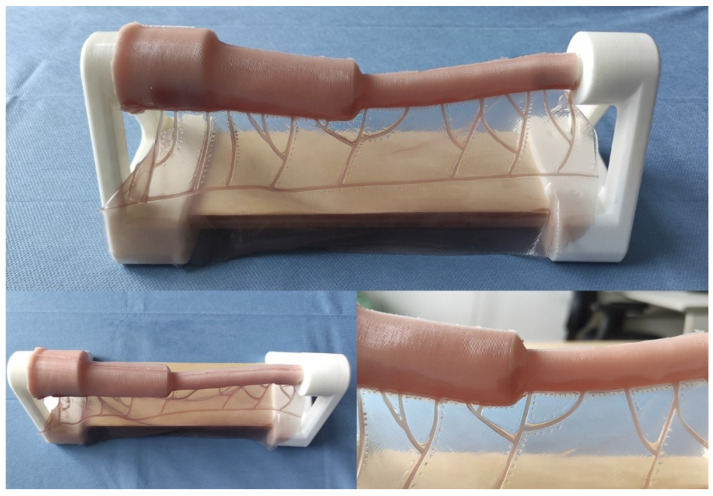
Appearance of the final model.

**Figure 3:**
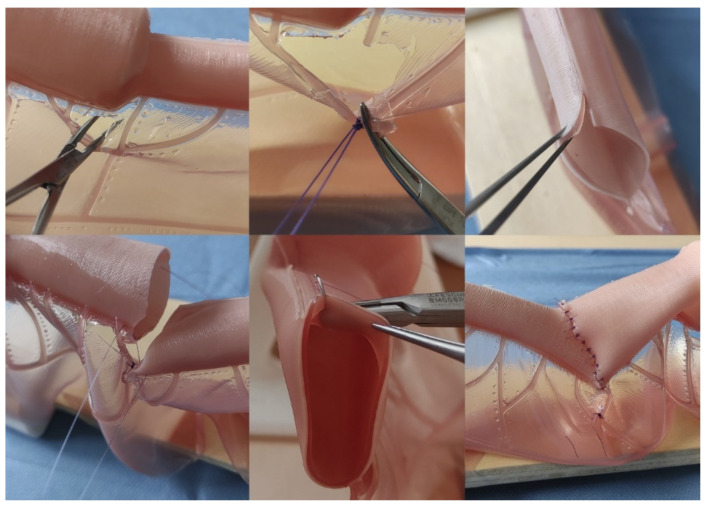
Representative images of the intestinal atresia open correction performance on the model. **Above Left, Center**: dissection and ligation of mesenteric vessels; **Above Right**: distal (atretic) intestinal end after performing anastomotic congruence maneuvers (Benson and Nixon). **Bottom Left**: lateral references before anastomosis; **Bottom Center**: detail showing the bilayer structure of the intestinal model and how the needle only goes through the external layer (seromuscular); **Bottom Right**: Final aspect after the procedure.

This model constitutes a low-cost and anatomically realistic representation of neonatal jejunoileal atresia with discordant ends, placed on a stand for optimal surgical technique positioning. The proximal intestinal segment (dilated) measures 12 cm in length and has a luminal diameter of 20 mm. It is composed of two platinum silicone layers of different hardness. The inner layer simulates the mucosal-muscular tissue and is 0.8 mm thick, while the outer layer simulates the serosa, is 0.3 mm thick, and is made of stiffer silicone. The distal intestinal segment (atretic) measures 12 cm in length and has a luminal diameter of 8 mm. It replicates the same layers and thicknesses of the proximal intestinal segment end. The simulator also includes a vascularized mesentery made of a thin silicone sheet, facilitating training in vascular control and bowel sectioning skills specific to this type of intervention (Figure 2 and Figure 3).

The complete model’s production time is 4 h, and the replacement of parts (silicone) takes 3.5 h. The production cost, including materials, labor, and indirect costs (electricity consumption), is EUR 45 for the complete model (including the stand) and EUR 25.21 for the model without the stand. This price is estimated for individual handmade production. An industrialized production process would lower costs and provide greater uniformity, making the product more competitive.

This project and the previously reported model of type III esophageal atresia [13] belong to the SIMUPED^®^ simulation development group.

### 2.4. Validation

A validation protocol was carried out using two groups of validators: experts (group 1) and non-experts (group 2). Group 1 comprised consultant pediatric surgeons who had performed the procedure on at least one previous occasion. Group 2 consisted of General or Pediatric Surgery residents in their second to fifth year of training. They had basic surgical skills but no specific training in neonatal surgical pathology or the surgical management of intestinal atresia.

Validation was conducted through a two-stage process. (1) First, an instructional video was presented to demonstrate the surgical procedure using the model (Supplementary Material S1). (2) Second, the procedure was performed under the direct visual supervision of two team collaborators (OEB and BPR) with continuous recording of the surgical field for reassessment. Participants did not see or manipulate the model before the validation procedure.

Specific questionnaires and checklists were developed to assess content, face, and construct validity. Each questionnaire included 10 items on content validity, 20 on face validity, and 21 on construct validity. All items were rated on a Likert scale from 1 (strongly disagree) to 4 (strongly agree).

### 2.5. Statistical Analysis

Continuous quantitative variables were expressed as mean (standard deviation). We used the Mann–Whitney U test to compare these variables. The statistical significance value was set at *p* = 0.05 (two-tailed). All analyses were performed in STATA 17.0 (StataCorp, LLC 4905 Lakeway Dr, College Station, TX 77845, USA)^®^.

## 3. Results

### 3.1. Construct Validity

The mean procedure time for the experts and non-experts groups was 41 (sd = 3.70) and 42 (sd = 5.15) minutes, respectively.

Two team collaborators completed a checklist regarding the construct validity questionnaire for all the experts (*n* = 11) and non-experts (*n* = 9). We found statistically significant differences in the proportion of participants who “Adequately calculates the section length of the antimesenteric border (Nixon technique)”, which was 100% in the expert group and 22.2% in the non-expert group (*p* = 0.01). Statistically significant differences were also found in the number of validators who completed the procedure within the established time, 90.9% in the expert group (*n* = 10) and 33% in the non-expert group (*n* = 3) (*p* = 0.02). Table 1 shows the comparison between groups for construct validity items.

### 3.2. Face Validity

All the experts (n = 11) responded to the face validity questionnaire. The best-rated items were those concerning the simulation of anastomotic congruence techniques (Benson and Nixon), with an average score of 3.6 out of 4. The worst-rated item was “the model reproduces the surgical dimensions of a neonatal abdominal field”, with a mean score of 2.3 out of 4. The mean score of the face validity questionnaire was 3.1 out of 4 (sd = 0.4). Table 2 shows the mean score of each item in the face validity questionnaire.

### 3.3. Content Validity

All the experts (n = 11) and non-experts (n = 9) responded to the content validity questionnaire. The best-rated item in the experts group was “This model helps the user understand the surgical technique”, with a mean score of 3.9 out of 4. The worst-rated item was “This model helps the user learn how to handle the neonatal bowel and mesenteric structures in a surgical context”, with a mean score of 2.7 out of 4. In the non-experts group, the best-rated items were “This model allows you to LEARN different surgical techniques”, “This model allows you to TRAIN different surgical techniques”, and “This model helps the user to be better prepared when performing corrective surgery for jejunoileal atresia in a neonate for the first time”, with a mean score of 3.6 out of 4. The worst-rated items were “This model allows to EVALUATE the user’s surgical technique” and “This model helps the user understand how intestinal tissue responds to being handled in surgery”, with a mean score of 3.1 out of 4. The mean score of the content validity questionnaire was 3.3 out of 4 for the experts (sd = 0.38) and 3.4 out of 4 for the non-experts (sd = 0.18). Table 3 shows the mean score of each item in the content validity questionnaire.

## 4. Discussion

In the present work, we designed, produced, and validated a low-cost and anatomically realistic model of neonatal intestinal atresia with eleven experts and nine non-experts.

Since the literature in this regard is scarce [16], the most challenging aspect in the design and development of this model was the search for precise references of the anatomical calibers and measures. Pediatric surgeons’ participation in this phase was essential. In this regard, the publication of precise anatomical calibers and measures of the different neonatal pathologies may contribute to developing more realistic models in the future.

Correcting neonatal intestinal atresia requires specific surgical maneuvers, such as anastomotic congruence techniques. This validation study showed the highest scores in the items related to these maneuvers, demonstrating that this model is valid for learning and training. In our experience, the design of a tubular structure simulating the bowel is complex. It requires a delicate balance between the tube maintaining structural integrity and collapsing: excessive stiffness in the tube is unnatural, and too little stiffness collapses the interior and makes the practice equally difficult to perform. In our case, we achieved a reasonably realistic situation. However, after the anastomotic congruence maneuvers (Benson and Nixon), two small silicone apexes in the distal end remained, and they had to be sectioned before anastomosis. Figure 3 (above, right) illustrates these apexes (one of them is gripped by the clamp). Future designs with more realistic materials may solve this minor problem. Likewise, in the future, this model may allow new anastomotic congruency techniques to be designed and trained before they are tested in animals and before they are applied to humans.

One of this model’s most outstanding and innovative elements is the mesentery. Although the characteristics of the mesentery obtained a relatively low score in the face validity questionnaire, the construct validity evaluation showed differences between the two groups (i.e., “Ligates the mesenteric vessels without grasping any of them with the forceps”, which showed a proportion of 63.6% in the experts group and 22.2% in the non-experts group, *p* = 0.09). Despite the lack of statistical significance (attributable to the study’s limited sample size), this difference suggests that the experts were more able to manipulate delicate tissues than non-experts. Mesenteric surgical principles in the neonate are an integral part of the corrective procedure for neonatal intestinal atresia (both because of the tissue delicacy and these patients’ hemodynamic lability). Therefore, implementing the mesentery in this model constitutes a novelty and opens the way to new design lines.

Although the current trend is towards the development of minimally invasive surgery (MIS), experience in intestinal atresia is limited. In this context, it seems crucial that future specialists acquire the essential surgical skills of open surgery before progressing to MIS. Nevertheless, this model could be introduced in a simulated neonatal abdominal box to train the MIS technique. 

Finally, we believe that the intestinal model we have produced (bilayer with differences in the hardness of each layer) allows for multiple types of intestinal suturing (seromuscular, total thickness …), which enriches the user’s training experience. We consider this a substantial improvement and difference from the existing precedent in the literature published by Takazawa et al. [16].

The construct validity showed exciting differences between groups on critical aspects of the surgical procedure (e.g., “Resects only the essential amount of affected intestine”, with 100% in the case of experts and 66.7% in the case of non-experts; *p* = 0.07). We attribute those items’ lack of statistical significance to the low sample size. The scarcity of pediatric surgeons and their broad geographical dispersion in Spain constituted essential difficulties when recruiting experts.

We believe using simulated models in Pediatric Surgery is promising for several reasons. The first is their low production cost and reproducibility. Animal models, which are expensive and may present some ethical conflicts, have an essential variability that may limit training conditions. Second, the required technology is available worldwide, which is particularly important in low- to middle-income countries. Third, it is easy to set up an individual practice.

Although this manuscript is confined to a pediatric surgical training model, simulation is relevant to all surgical specialties. Recent publications concerning training models in General Surgery [20], Obstetrics [21], and Vascular Surgery [22], among other specialties, attest to this. In our view, creating collaborative networks to share advances in the design and development of these models between specialties and to contribute to better tissue engineering and simulation is essential.

Lastly, new technical resources such as 3D simulation and virtual reality show enormous potential for surgical skills training. Applications such as Lap Mentor^®^ or LapSim^®^ have demonstrated enormous potential in acquiring surgical skills. These devices present a considerable advantage in haptic feedback and simulation of complex scenarios that cannot be easily simulated in synthetic or animal models [23]. Also, a significant advantage of these devices is the absence of consumable consumption and the possibility of reuse at no additional cost. Sustainability and ecology, which have been scarcely considered in surgery until now, are beginning to play a relevant role, and this aspect should be considered for future studies.

Concerning the strengths and limitations of this study, we acknowledge that the small sample size of both groups represents a significant limitation of this study. Furthermore, more complex validation systems (such as pressure sensors or leakage tests) would have provided more objective information. Also, the fact that the team collaborators who completed the construct validity questionnaire were not blinded to the type of participant (expert or non-expert) may have affected the results. Lastly, it should be considered that animal models present intrinsic advantages in terms of training that are not easily replicable, such as the acquisition of hemostasis skills. These represent a challenge for the future in this line of research. On the other hand, the methodological rigor in the study’s design and performance represents this work’s main strength.

In conclusion, we designed, created, and validated a low-cost, realistic model for training neonatal intestinal atresia open surgery. However, further studies with larger sample sizes and external validators blinded to the type of participants are needed before drawing definitive conclusions. Because simulators in Pediatric Surgery may contribute to better global care of children, especially neonates, this line of research should become a priority.

## Figures and Tables

**Table 1:** Construct validity evaluation.

Item	Experts (n = 11)	Non-Experts (n = 9)	*p*-Value
Decides to ligate the mesenteric vessels before sectioning (% Yes)	72.7	55.6	0.64
Ligates the mesenteric vessels without tearing them (% Yes)	45.5	22.2	0.37
Ligates the mesenteric vessels without grasping any of them with the forceps (% Yes)	63.6	22.2	0.09
Does not produce damage to mesenteric vessels during the whole process (% Yes)	72.7	44.4	0.36
Correctly identifies the mesenteric section area and vascular boundaries of the intestinal segment to be resected (% Yes)	81.8	77.8	0.99
Sections bowel accurately, without deviation (% Yes)	81.8	33.3	0.07
Resects only the essential amount of affected intestine (<2 cm from the beginning of the healthy intestine in each segment) (% Yes)	100	66.7	0.07
Adequately spatulates intestinal loop (Benson technique) (% Yes)	72.7	33.3	0.18
Adequately calculates the section length of the antimesenteric border (Nixon technique) (% Yes)	**100**	**22.2**	**0.01**
Start anastomosis with side sutures and reference them (% Yes)	90.0	100	0.99
In general, sutures are seromuscular (% Yes)	100	88.9	0.45
Sutures are generally equidistant from each other (% Yes)	100	88.9	0.45
In general, the entry points of the suture in relation to the free margin are equidistant (% Yes)	100	88.9	0.45
In general, hand-made knots are performed correctly (“surgeon’s knot”) (% Yes)	100	100	0.99
In general, knots are performed correctly using the Mayo holder (% Yes)	100	100	0.99
Does not leave overlapping anastomotic edges (% Yes)	36.4	22.2	0.64
Does not tear serosa (% Yes)	45.5	66.7	0.41
Correctly repositions lateral references before intestinal rotation (% Yes)	81.8	66.7	0.62
Adequately rotates the anastomosis without damaging the mesentery or the bowel (% Yes)	63.6	44.4	0.65
Number of sutures employed (average)	3	2.7	0.25
Completes procedure in <45 min (% Yes)	**90.9**	**33.3**	**0.02**
Average time to complete the procedure (only takes into account those who completed the procedure) (minutes)	41 (3.70) (n = 10) *	42 (5.15) (n = 3) *	0.61

*: Mean (standard deviation).

**Table 2:** Face validity questionnaire.

Item	Mean Score (Experts) N = 11
The intestinal diameter of the model resembles that of neonatal intestinal atresia.	2.7
The intestinal thickness of the model resembles that of neonatal intestinal atresia.	3.0
The mesentery resembles that of a real neonatal intestine.	2.4
Mesentery vessels are adequately represented.	3.2
The layers of the model realistically simulate a real neonatal intestine.	3.4
Visually, the model material resembles a real neonatal intestine.	3.5
The model’s feel (wet and slippery) resembles a real neonatal intestine.	2.8
The texture and consistency of the model when the suture needle passes through it is similar to that of a real neonatal intestine.	3.3
The sensation of suturing the model’s strands together is similar to that of a real neonatal intestine.	3.1
The model reproduces the surgical dimensions of a neonatal abdominal field.	2.3
The spatial positioning of the model resembles that of a surgical field.	2.5
The model allows the mobility of the structures to be similar to reality.	2.9
In general, the execution of the surgical technique for the correction of neonatal intestinal atresia on the model resembles reality	3.0
This model is USEFUL for LEARNING the surgical technique for correcting intestinal atresia.	3.6
This model is USEFUL for TRAINING the surgical technique for correcting intestinal atresia.	3.5
This model allows realistic simulation of the Benson anastomotic congruency technique (spatulation of the distal anastomotic end).	3.6
This model allows a realistic simulation of Nixon’s anastomotic congruence technique (longitudinal opening of the antimesenteric edge of the distal anastomotic end).	3.6
This model allows a realistic simulation of an end-to-end intestinal anastomosis with discordant caliber ends.	3.5
This model allows the acquisition of transferable surgical skills to the actual surgical field.	3.5
This model realistically reproduces the level of difficulty of the procedure.	3.1
**MEAN SCORE**	**3.1**

Each item was evaluated on a 4-point Likert scale (1: totally disagree–4: totally agree).

**Table 3:** Content validity questionnaire.

Item	Mean Score (Experts) N = 11	Mean Score (Non-Experts) N = 9	*p*-Value
This model helps the user to understand the surgical technique.	3.9	3.4	0.17
This model helps the user to understand the spatial arrangement of the intestine and mesentery in a neonate.	3.3	3.4	0.74
This model helps the user learn how to handle the neonatal bowel and mesenteric structures in a surgical context.	2.7	3.2	0.33
This model helps the user understand how intestinal tissue responds to being handled in surgery.	3.0	3.1	0.74
This model allows you to LEARN different surgical techniques.	3.6	3.6	0.88
This model allows TRAINING different surgical techniques.	3.5	3.6	0.84
This model allows to EVALUATE the user’s surgical technique.	2.9	3.1	0.56
This model allows users to measure their ability to perform corrective surgery for neonatal intestinal atresia.	3.1	3.2	0.67
This model helps the user to be better prepared when performing corrective surgery for intestinal atresia in a neonate for the first time.	3.5	3.6	0.93
This model increases the user’s confidence before performing corrective surgery for intestinal atresia in a neonate.	3.5	3.4	0.59
**MEAN SCORE**	3.3 (0.38) *	3.4 (0.18) *	**0.74**

Each item was evaluated on a 4-point Likert scale (1: totally disagree–4: totally agree). *: Mean (standard deviation).

## Data Availability

The data used to carry out this study are available upon request from the review authors due to personal reason.

## Supplementary Materials

The following supporting information can be downloaded at: https://www.mdpi.com/article/10.3390/children11091109/s1, Video demonstration of the surgical correction of neonatal intestinal atresia in the model.

## Industrial Property

The present model is under industrial property registration and protection under the code U202330721.
